# Editorial: Motor Priming for Motor Recovery: Neural Mechanisms and Clinical Perspectives

**DOI:** 10.3389/fneur.2017.00448

**Published:** 2017-09-04

**Authors:** Sangeetha Madhavan, Mary Ellen Stoykov

**Affiliations:** ^1^University of Illinois at Chicago, Chicago, IL, United States; ^2^Shirley Ryan Ability Lab, Chicago, IL, United States

**Keywords:** priming, neuroplasticity, recovery, brain, movement

**Editorial on the Research Topic**

**Motor Priming for Motor Recovery: Neural Mechanisms and Clinical Perspectives**

The Oxford dictionary defines the term priming as “a substance that prepares something for use or action.” In this special issue, we define motor priming as a technique, experience, or activity targeting the motor cortex resulting in subsequent changes in motor behavior. Inadequate functional recovery after neural damage is a persisting burden for many, and this insufficiency highlights the need for new neurorehabilitation paradigms that facilitate the capacity of the brain to learn and recover. The concept of motor priming has gained importance in the last decade. Numerous motor priming paradigms have emerged to demonstrate success to improve functional recovery after injury. Some of the successful priming paradigms that have shown to alter motor behavior and are easily implementable in clinical practice include non-invasive brain stimulation, movement priming, motor imagery, and sensory priming. The full clinical impact of these priming paradigms has not yet been realized due to limited evidence regarding neural mechanisms, safety and effectiveness, dosage, individualization of parameters, identification of the appropriate therapies that need to be provided in combination with the priming technique, and the vital time window to maximize the effectiveness of priming. In this special issue, four manuscripts address critical questions that will enhance our understanding of motor priming paradigms and attempt to bridge the gap between neurophysiology and clinical implementation.

In their study, “Non-Invasive Brain Stimulation to Enhance Upper Limb Motor Practice Poststroke: A Model for Selection of Cortical Site,” Harris-Love and Harrington elegantly address the extremely important issue of individualizing brain stimulation for upper limb stroke recovery. Many brain stimulation techniques show high interindividual variability and low reliability as the “one-size-for-all” does not fit the vast heterogeneity in recovery observed in stroke survivors. In this article, the authors propose a novel framework that personalizes the application of non-invasive brain stimulation based on understanding of the structural anatomy, neural connectivity, and task attributes. They further provide experimental support for this idea with data from severely impaired stroke survivors that validate the proposed framework.

The issue of heterogeneity poststroke is also addressed by Lefebvre and Liew in “Anatomical Parameters of tDCS to modulate the motor system after stroke: A review.” These authors discuss the variability in research using tDCS for the poststroke population. According to the authors, the most likely sources of variability include the heterogeneity of poststroke populations and the experimental paradigms. Individually based variability of results could be related to various factors including: (1) molecular factors such as baseline measures of GABA, levels of dopamine receptor activity, and propensity of brain-derived neurotropic factor expression; (2) time poststroke, (3) lesion location; (4) type of stroke; and (5) level of poststroke motor impairment. Variability related to experimental paradigms include the timing of the stimulation (pre- or post-training), the experimental task, and whether the protocol emphasizes motor performance (a temporary change in motor ability) or motor learning based (more permanent change in motor ability). Finally, the numerous possibilities of electrode placement, neural targets, and the different setups (monocephalic versus bi-hemispheric) add further complexity. For future work with the poststroke population, the authors suggest that tDCS experimental paradigms explore individualized neural targets determined by neuronavigation.

In another exciting study in this issue, Estes et al. tackle the timely topic of spinal reflex excitability modulated by motor priming in individuals with spinal cord injury. The authors choose to test four non-pharmacological interventions: stretching, continuous passive motion, transcranial direct current stimulation, and transcutaneous spinal cord stimulation to reduce spasticity. Three out of four techniques were associated with reduction in spasticity immediately after treatment, to an extent comparable to pharmacological approaches. These priming approaches provide a low-cost and low-risk alternative to anti-spasticity medications.

In another clinical study in individuals with spinal cord injury, Gomes-Osman et al. examined effects of two different approaches to priming. Participants were randomized to either peripheral nerve stimulation (PNS) plus functional task practice, PNS alone, or conventional exercise therapy. The findings were unexpected. There was no change in somatosensory function or power grip strength in any of the groups. Interestingly, all of the interventions produced changes in precision grip of the weaker hand following training. However, only PNS plus functional task practice improved precision grip in both hands. The authors found that baseline corticospinal excitability were significantly correlated to changes in precision grip strength of the weaker hand. The lack of change in grip strength in any of the groups was surprising. Previous evidence suggests, however, that the corticomotor system is more strongly activated during precision grip as compared to power grip, and the authors suggest that interventions targeting the corticomotor system (i.e., various priming methods) may more strongly effect precision grip.

Overall, this special issue brings together an array of original research articles and reviews that further enhance our understanding of motor priming for motor recovery with an emphasis on neural mechanisms and clinical implementation. We hope that the studies presented encourage future studies on motor priming paradigms to optimize the potential for functional recovery in the neurologically disadvantaged population, and further our understanding of neuroplasticity after injury.

## Author Contributions

SM and MS have made a substantial, direct, and intellectual contribution to the work and approved it for publication.

## Conflict of Interest Statement

The authors declare that the research was conducted in the absence of any commercial or financial relationships that could be construed as a potential conflict of interest.

